# Bilateral Pseudomonas endophthalmitis after immediately sequential bilateral cataract surgery: primum non nocere

**DOI:** 10.5935/0004-2749.20200073

**Published:** 2020

**Authors:** Steve A. Arshinoff, Charles Claoué, Cyres Mehta, Bjorn Johanssen

**Affiliations:** 1 Executive members, International Society of Bilateral Cataract Surgeons, Toronto, CA; 2 Executive members, International Society of Bilateral Cataract Surgeons, London, UK; 3 Executive members, International Society of Bilateral Cataract Surgeons, Linkoping, SE; 4 Executive members, International Society of Bilateral Cataract Surgeons, Mumbai, IN

Dear Editor,

We read with great interest and significant concern the recent letter by Ting et al. concerning the report of bilateral *Pseudomonas* postoperative endophthalmitis (POE) published in your journal in 2018, about which we submitted a letter published in 2019, along with a response from the author Mota, who also responded to the current letter by Ting et al.^(1-3)^. We appreciate the opportunity to respond further to the discussion.

We thank Dr. Ting et al.^(1)^ for agreeing with our concern that republication of a 3-year-old case from another medical journal, switching photographs, and without reference to the first publication is not the accepted expectation for original case reports. According to the Committee on Publication Ethics, such conduct is frowned upon as unethical and obviously can distort the available body of evidence and the weighting of the impact of reported facts and circumstances^(4)^.

Ting et al. state that the incidence of POE quoted by Mota (~0.05%) appears to be unrealistic based on their own experience and referenced literature evidence, and they quote a higher incidence due to leaky clear corneal incisions. The issues of clear corneal incisions were summarized and resolved by the American Society of Cataract and Refractive Surgery (ASCRS) white paper of 2006^(5)^. Although leaky incisions may predispose to postoperative ingress of bacteria into the eye, well-constructed incisions seal and do not. Over the past 20 years, infection rates have gradually decreased due to multiple enhancements in surgical procedures and are currently quoted in the range of 0.04%, even when a significant portion of the cases included in the source of this reference, the US Intelligent Research in Sight (IRIS) study (2013-2017 cases, including 5,401,686 eyes), may not have received intracameral antibiotics (IC)^(6)^.

Ting et al. express disbelief in intracameral antibiotics and refer to “large studies in Europe and smaller studies out of North America, all of which were retrospective.” They overlook the landmark European Society of Cataract & Refractive Surgeons (ESCRS) prospective randomized international multicenter clinical study that confirmed previous Swedish reports on how IC lowered the infection rates dramatically by 80%^(7)^. Subsequent clinical studies have provided further confirmation and now include approximately 10 million investigated eyes. By not citing the body of evidence properly, we are significantly concerned that Ting et al. provide readers a skewed perception of the efficacy of IC in POE prophylaxis.

None of this addresses the issue of immediately sequential bilateral cataract surgery (ISBCS) versus delayed sequential bilateral cataract surgery (DSBCS). The incidence of POE reported in our study of infection after ISBCS, referenced in our first letter, was 1:14,352 (0.007%) in patients who received prophylactic IC, the lowest POE rate ever reported in any study to date. That study was published in 2011, and our reported POE rate has, if anything, decreased with ISBCS since then.

Ting et al. then go on to finalize their comments by saying that both they, individually, and the Royal Australian and New Zealand College of Ophthalmologists are opposed to ISBCS, quoting “*primum non nocere*,” but appear not to fully comprehend its meaning or origin:

“*Primum non nocere*” is indeed Latin but is generally attributed to Hippocrates and therefore should be in Greek. We believe that Ting et al. should be reminded of parts of the Hippocratic Oath that remain as true today as when first enunciated:

“I will use treatment to help the sick according to my ability and judgment, but never with a view to injury and wrong-doing.… Into whatsoever houses I enter, I will enter to help the sick, and I will abstain from all intentional wrong-doing and harm, especially from abusing the bodies of man or woman.”

Hippocrates clearly understood and respected the differences of skill and opinion using the phrase “my ability and judgement”. To imply, as Ting et al. do, that the large body of mature, reflective, evidence-based opinion is intentionally setting out to create bilateral simultaneous endophthalmitis is simply nonsense. The Medical Board of Australia in their Good Medial Practice: A Code of Conduct at 4.2 requires Australian doctors to respect others’ opinions.

Those who practice immediately sequential bilateral cataract surgery (ISBCS) to a high standard respect the right of those with an alternative opinion and are entitled to the same. In 2009, the International Society of Bilateral Cataract Surgeons (*i*SBCS) published a document, referenced in our first letter, concerning optimal practice. We have had numerous requests to assist others in transitioning to ISBCS, because reducing the number of patients coming in for surgery by 50%, and reducing their needed frequency of return by 50%, should reduce their risk of hospital-acquired infections, and now particularly COVID-19. In this context, we paraphrase Jon Bolger’s astute comment of 2008 concerning then-current risks as “No one will state that ISBCS has no risks, nor that the risk of bilateral endophthalmitis is zero. We currently (2008) have the ability to reduce that risk to about 1:1 000 000, or less, which is considerably less than the risk that patients take traveling to the extra visits required for two unilateral cataract surgical procedures compared to ISBCS. We may perhaps see one devastating bilateral endophthalmitis after ISBCS, optimally performed, for every 3 traffic deaths we avoid by performing ISBCS (based on U.K. traffic mortality data per kilometer driven). In life everything has risk. We simply should intelligently choose the risks we will face and try to control them, rather than avoid one fearful risk and by so doing run headlong into a much worse, more common one.”^(7)^

With respect to the response of Mota to Ting et al., we frankly feel that, without prejudice, it added little to the discussion. Since the report of this case (twice), there has been another case of bilateral POE after ISBCS from Mexico^(9)^. In both the reported Mexican cases, the authors claim that they know nothing about the actual events of the original surgery and whether proper sterility and right to left procedural isolation precautions were followed or not. There have been less than 10 cases of bilateral POE reported globally over the past 40 years. The World Health Organization (WHO) estimates that roughly 20 million cataract surgeries are performed globally per year, suggesting that the incidence of bilateral POE is extremely low.


Sincerely, Steve A. Arshinoff MD, Toronto, Ontario, Canada Charles
Claoué MD, London, UK. Bjorn Johanssen MD, Linkoping, Sweden.
Cyres Mehta MD, Mumbai, India Executive members, International Society of
Bilateral Cataract Surgeons

