# Supplementary material for: Bilateral Pseudomonas endophthalmitis after immediately sequential bilateral cataract surgery: primum non nocere
Source: Arq Bras Oftalmol. 2020 Jul-Aug;83(4):346–9. doi: 10.5935/0004-2749.20200073 (PMC11826598; doi:10.5935/0004-2749.20200073)
Supplement: Related texts

# Reply to Dr. Arshinoff´s letter to ABO editor

**Authors:** Sergio Hernandez-Da Mota

**Affiliations:** 1 Retina Service, Clinica David, Unidad Oftalmologica, Morelia, Mexico

Dear editor:

We read with great interest the comments of Dr. Arshinoff et al. regarding our case report as well as the letter of Dr. Ting et al. to the editor previously published in ABO journal^(1,2)^_._

We reiterate that our intention in reporting this case was by no means mere sensationalism or disregarding ethical publication issues, as pointed out in our previous reply letters^(2,3)^.

We agree that with the advent of postoperative endophthalmitis (POE) prophylaxis and the improvements in surgical techniques, the incidence of POE has dramatically decreased^(4)^.

There is little doubt that immediately sequential bilateral cataract surgery (ISBCS) has advantages over delayed sequential bilateral cataract surgery (DSBCS)^(5)^. Therefore, many cataract surgeons operating under optimal conditions should definitely consider it as an alternative technique in patients with bilateral cataract.

Aside from the ongoing POE debate with both ISBCS and DSBCS techniques^(5)^, there are other related issues that we believe should also be addressed. Optimal perioperative and intraoperative conditions such as rigorous sterility protocols may be nonexistent in several centers that perform cataract surgery, primarily in third world countries. Although it is imperative to improve these conditions, this might not be feasible, at least in the short term. Noncompliance with operating room regulations, disregard of proper sterility guidelines^(6)^, heterogeneity in the standard of care of patients among hospitals, lack of sanitary surveillance by health authorities, and training of operating room personnel are some of the reasons that could explain suboptimal intraoperative and perioperative conditions.

These suboptimal conditions may increase the likelihood of POE, in both ISBCS and DSBCS. Nonetheless, several dissatisfied patients with unilateral POE who have not undergone surgery in the fellow eye would, for instance, have the opportunity to seek attention in another facility that has better standard of care and sterility protocols, thus probably and significantly decreasing the likelihood of POE in the other eye.

Under this scenario, DSBCS might still be a reasonable and safer option than ISBCS for patients in countries such as Mexico. Hence, these considerations may limit the widespread implementation of ISBCS for our cataract patients, until a high standard of sterility protocols and care is reached, in the vast majority of cataract surgery facilities in our country and in other underdeveloped countries around the world.

Finally, we acknowledge the world leadership and pioneering of Dr. Arshinoff et al. in the field of ISBCS. We appreciate their interest, insights, and remarks about our case report and this hot topic.


Sincerely, Sergio Hernandez-Da Mota


## References

[1] Ting ER, Lee BW, Jiang IW, Agar A, Francis IC. (2020). Blindness from bilateral pseudomonas endophthalmitis
following bilateral simultaneous cataract surgery: Primum non nocere.
Letter. Arq Bras Oftalmol.

[2] Mota S. (2018). Pseudomonas aeruginosa-induced bilateral endophthalmitis
after bilateral simultaneous cataract surgery: case
report. Arq Bras Oftalmol.

[3] Arshinoff SA, Claoué C, Mehta C, Johanssen B. (2019). Bilateral pseudomonas endophalmitis after immediately
sequential bilateral cataract surgery. Arq Bras Oftalmol.

[4] Endophthalmitis Study Group, European Society of Cataract &
Refractive Surgeons (2007). Prophylaxis of postoperative endophthalmitis following
cataract surgery: results of the ESCRS multicenter study and
identification of risk factors. J Cataract Refract Surg.

[5] Kessel L, Andresen J, Erngaard D, Flesner P, Tendal B, Hjortdal J. (2015). Immediate Sequential Bilateral Cataract Surgery: a systematic
review and meta-analysis. J Ophthalmol.

[6] International Society of Bilateral Cataract Surgeons
(ISBCS) (2009). General principles for excellence.
